# Persistent Lung Injury and Prothrombotic State in Long COVID

**DOI:** 10.3389/fimmu.2022.862522

**Published:** 2022-04-07

**Authors:** Mengqi Xiang, Haijiao Jing, Chengyue Wang, Valerie A. Novakovic, Jialan Shi

**Affiliations:** ^1^ Department of Hematology, First Affiliated Hospital of Harbin Medical University, Harbin Medical University, Harbin, China; ^2^ Department of Research, Veterans Affairs Boston Healthcare System, Harvard Medical School, Boston, MA, United States; ^3^ Department of Medical Oncology, Dana-Farber Cancer Institute, Harvard Medical School, Boston, MA, United States

**Keywords:** COVID-19, long COVID, thrombosis, phosphatidylserine, therapy, anticoagulation

## Abstract

Lung injury may persist during the recovery period of COVID-19 as shown through imaging, six-minute walk, and lung function tests. The pathophysiological mechanisms leading to long COVID have not been adequately explained. Our aim is to investigate the basis of pulmonary susceptibility during sequelae and the possibility that prothrombotic states may influence long-term pulmonary symptoms of COVID-19. The patient’s lungs remain vulnerable during the recovery stage due to persistent shedding of the virus, the inflammatory environment, the prothrombotic state, and injury and subsequent repair of the blood-air barrier. The transformation of inflammation to proliferation and fibrosis, hypoxia-involved vascular remodeling, vascular endothelial cell damage, phosphatidylserine-involved hypercoagulability, and continuous changes in serological markers all contribute to post-discharge lung injury. Considering the important role of microthrombus and arteriovenous thrombus in the process of pulmonary functional lesions to organic lesions, we further study the possibility that prothrombotic states, including pulmonary vascular endothelial cell activation and hypercoagulability, may affect long-term pulmonary symptoms in long COVID. Early use of combined anticoagulant and antiplatelet therapy is a promising approach to reduce the incidence of pulmonary sequelae. Essentially, early treatment can block the occurrence of thrombotic events. Because impeded pulmonary circulation causes large pressure imbalances over the alveolar membrane leading to the infiltration of plasma into the alveolar cavity, inhibition of thrombotic events can prevent pulmonary hypertension, formation of lung hyaline membranes, and lung consolidation.

## Introduction

While the majority of patients with coronavirus disease 2019 (COVID-19) will develop only mild, self-limited illness, up to 20% will progress to a more serious form, including severe pneumonia, acute respiratory distress syndrome (ARDS), and pulmonary fibrosis (1–8). The potential risk of pulmonary impairment and parenchymal fibrosis in long COVID is of particular concern (9–13), and studies of multiple treatment options for COVID-19 do not consider their effects on subsequent risk and progression of long-term COVID-19 symptoms (14). Multiple mechanisms of lung injury in COVID-19 patients have been tentatively described, but the long-term pathogenicity of SARS-CoV-2 in discharged patients remains unclear. It has been reported that the consequences of severe COVID-19 are similar to those of severe acute respiratory syndrome (SARS) and Middle East respiratory syndrome (MERS) in terms of clinical sequelae, respiratory function, mental illness, and health-related quality of life (15–17). After infection, virus-induced immunopathological events are believed to be responsible for the pulmonary manifestations of SARS and MERS. Specifically, the virus replicates rapidly, infects type II alveolar epithelial cells and vascular endothelial cells, and increases the production of proinflammatory cytokines and chemokines. These, in turn, recruit fibroblasts and induce their differentiation into myofibroblasts, resulting in impaired O_2_ and CO_2_ exchange (18). In addition, viral antagonism and delayed interferon responses further aggravate inflammation (19, 20).

When SARS-CoV-2 replicates in large numbers, immune cells and inflammatory mediators respond strongly, forming cytokine storms and damaging alveolar structures. The virus invades vascular endotheliocytes from the blood-air barrier. As the disease progresses, endothelial dysfunction leads to more rigid and therefore vulnerable pulmonary vessels. Vascular endothelium expresses more protease activated receptor 1, tissue factor (TF), P-selectin and phosphatidylserine (PS) on the membrane surfaces, releasing microparticles, von Willebrand Factor (vWF), and factor VIII (21). These alterations, together with soluble thrombomodulin (sTM) and increased surface chemokines, causes platelet overactivation and thrombosis (22). With the enhanced permeability of the alveolar membrane, the pulmonary edema causes further hypoxemia and deterioration (23, 24). Pulmonary (micro)thrombus is key to severe hypoxemia, multiple organ dysfunction, and prolonged COVID-19 syndrome (25–33). Microthrombi can block microvessels in the alveolar capillaries, making it difficult for red blood cells to pass through. Slow blood flow and local congestion lead to elevated pulmonary capillary pressure and then to pulmonary hypertension (4). The pressure difference between the two sides of the blood-air barrier increases, while severe inflammation causes diffuse alveolar damage, resulting in the inability of the alveolar membrane to maintain normal permeability (34). Various components in the blood, including macromolecules (mainly albumin and globulin), enter the alveolar cavity. This fluid in the alveolar cavity then induces aggravated dyspnea (35–37). The alveolar liquid evaporates under airflow action, leaving behind plasma proteins and necrotic alveolar epithelial debris to form a transparent membrane, leading to lung consolidation (3–6, 38, 39). Although hypoxemia results from a combination of many mechanisms, the amplifying effects of hypoxia promote the exacerbation of cytokine storms, endothelial injury and thrombosis (23, 24). In long COVID, patients often show substandard six-minute walk test (6mWT), abnormal chest imaging findings (such as bilateral interstitial infiltration, ground-glass opacity (GGO), and fibrosis), and lung diffusing capacity for carbon monoxide (DLCO) < 80%, all indicating persistent lung damage (11–13, 40). In a follow-up study of 113 COVID-19 patients with ARDS, 55% reported dyspnea eight months after diagnosis. Adjusted for age, more than 50% of patients who undertook a 6mWT reached less than 80% of the theoretical distance. Abnormal chest radiographs were reported in 49% of cases, with bilateral interstitial infiltration predominating (87.5%). Chest computerized tomography (CT) scans showing GGO (55%) and fibrosis (19%) were common. Additionally, DLCO was less than 80% in 77.8% of patients (41).

The frequently reported pulmonary arterial, venous, and capillary thrombotic events at autopsy suggest that the transformation of stable vascular endothelial cells to the prothrombotic state is not negligible (8, 25, 27–29, 35, 38–47). We therefore hypothesize that the effects of thrombosis may persist long after the patient has met the criteria for discharge (27). In the recovery stage, it is worth considering whether the prothrombotic status is neglected in patients without thrombotic complications and whether procoagulant factors (such as PS exposure) return to normal in patients with thrombosis. We will investigate pulmonary susceptibility in long COVID and the possibility that prothrombotic states may influence long-term pulmonary symptoms.

## Continuous Shedding of the Virus

Tarhini et al. reported cases of severely immunocompromised COVID-19 patients shedding infectious virus up to four months following symptom onset. In one instance, they reported a single persistent infection with high load culture-positive virus and positive reverse transcription polymerase chain reaction (RT-PCR) on day 103 (27 days after readmission). This study also included a discharged patient who developed post-COVID pneumonia with active virus replication in the lower respiratory tract and finally developed a double infection after second admission (48). The immune system can be suppressed or even depleted by fighting off the increasing viral load (23, 49). Therefore, even if patients have no compromised immune system before SARS-CoV-2 infection, they may show similar symptoms during the disease. In these cases, the virus may retain the ability to transmit over time, as evidenced by positive viral cultures. A variety of scenarios (such as asymptomatic carrier, symptom resolution, or secondary infection) allow for prolonged infectious virus emission (12, 50). In a cross-sectional study, 10 of 60 discharged COVID-19 patients tested positive for SARS-CoV-2 by RT-PCR 4-24 days after discharge. Since all discharged patients were instructed to stay at home and local cases were rare, the researchers assumed that the positive result was persistent virus shedding rather than reinfection (51). Viral persistence is associated with more extensive tissue invasion and worse recovery outcomes. Another study found that the average shedding time of the virus was 19 days in asymptomatic patients, ranging from 6-45 days. While circulating antibodies to other coronaviruses (such as SARS-CoV or MERS-CoV) have been shown to last for at least a year, antibodies to SARS-CoV-2 wane relatively quickly. In both the asymptomatic and symptomatic groups, IgG levels fell by more than 70% in more than 90% of cases during the early recovery period (8 weeks after discharge) (52). In a cohort study, the positive serum rate and median titer of neutralizing antibodies were significantly lower in the convalescent follow-up than during acute infection (12). A report assessed 30,082 patients with mild-to-moderate COVID-19 indicated that antibody titers remained stable at three months but declined slightly at the 5-month time point (49). There is evidence that the antigenicity of SARS-CoV-2 spike protein changes. Spike of amino acid substitutions and deletions impact neutralizing antibodies and the variants are resistant to antibody-mediated immunity elicited by vaccines (53). Therefore, the risk of reinfection should be monitored, especially in patients with prolonged viral shedding (54). SARS-CoV-2 is likely to persist in certain tissues to drive chronic symptoms. In a follow-up trial, approximately 4-6 months after diagnosis, positive SARS-CoV-2 ribonucleic acid (RNA) was detected in olfactory mucosa samples from four patients with negative nasopharyngeal swabs for SARS-CoV-2 RNA (55).

## Pulmonary Vulnerability

### Early Stage

**Figure 1:**
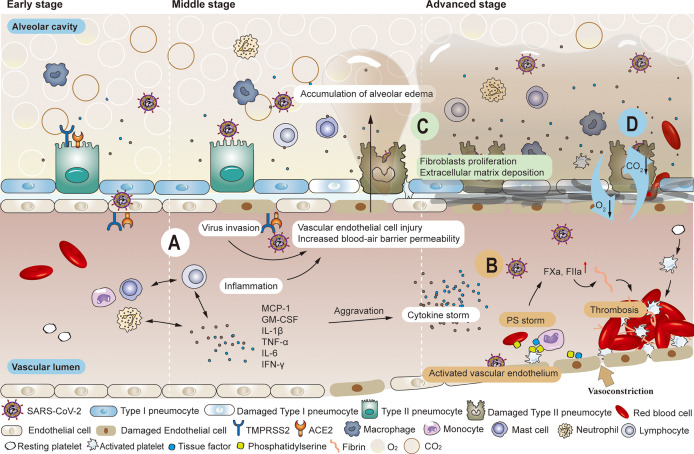
Common pathological changes in the lungs of patients with COVID-19. **(A)** SARS-COV-2 enters local alveolar type II cells along the airway, replicates, and damages the targeted cells. Various immune cells are mobilized and activated wherever the virus goes, inducing the release of inflammatory factors such as MCP-1, GM-CSF, IL-1β, TNF-α, IL-6, IFN-γ, etc. The inflammatory response produced in killing the virus also leads to the injury of alveolar type I and type II cells. When high viral load accompanies the inflammatory response, the air-blood barrier is destroyed on the alveolar side. The virus has the opportunity to invade vascular endothelial cells by crossing the local air-blood barrier, and strong cytokine responses can also spread to vascular endothelial cells. As the permeability of the blood-air barrier increases, blood components enter the alveolar cavity, forming pulmonary edema. **(B)** Significantly enhanced thrombin and elevated levels of endothelial cell biomarkers (vWF: Ag, vWFpp, FVIII, and sTM) were observed in the convalescence period. Impaired pulmonary vascular endothelium can cause uncontrolled activation of coagulation cascades, further leading to vascular thrombosis or fatal pulmonary fibrosis. **(C)** The initial response to the destruction of the alveolar epithelial-endothelial barrier is edematous infiltration in the alveoli and interstitial portion, followed by proliferation as the alveolar barrier is rebuilt by removing exudate. Extracellular matrix deposition occurs. Fibroblasts migrate and transform into muscle cells. **(D)** The damaged blood-air barrier, impaired pulmonary blood perfusion, reduced effective volume of alveolar cavities and the appearance of fibrosis all lead to the obstruction of the exchange of oxygen and carbon dioxide.

SARS-CoV-2 enters local alveolar type II cells along the airway, replicates, and damages the targeted cells. Inflammatory mediators are produced when sentinel cells (pulmonary macrophages in the lung interstitium) surrounding the injured tissue recognize the damaged target cells. These mediators trigger neutrophils and monocytes in the blood circulation to migrate to the injured site under the action of chemokines. Various immune cells are mobilized and activated wherever the virus goes, inducing the release of inflammatory factors such as monocyte chemoattractant protein-1 (MCP-1), granulocyte-macrophage colony-stimulating factor (GM-CSF), interleukin-1β (IL-1β), tumor necrosis factor-α (TNF-α), interleukin 6 (IL-6), interferon-γ (IFN-γ), etc (56). Leukocytes, while necessary to phagocytose inflammatory substances, can also release lysosomal enzymes, reactive oxygen species, and free radicals into the extracellular stroma, damaging normal alveolar type I and II cells. Another function of leukocytes is to recognize membrane expression of PS, an ‘eat me’ signal for macrophages lest more extensive pro-coagulant surfaces appear (57, 58). Ideally, SARS-CoV-2 is gradually cleared by three defensive walls consisting of airway secretions, ciliary oscillations, and innate and acquired immune cells. Even if local ciliated goblet cells, mucous goblet cells, and alveolar epithelial cells are damaged, mild conditions will not evolve into persistent symptoms, and virus numbers may quickly start to decrease. The damaged target cells in the airway can be repaired by the proliferation and differentiation of basal cells. Still, alveolar cells are difficult to regenerate, which is often the precursor to long-term symptoms (59). The virus has the opportunity to invade vascular endothelial cells by crossing the local air-blood barrier (60, 61) (**Figure 1A**). However, the extent of the damage to the local vascular endothelial cells is difficult to predict in early stage. Nevertheless, these impaired vascular endothelial cells often serve as the initial site of thrombosis. At this time, activated vascular endothelial cells are often overlooked, but they are the vulnerable basis of long COVID after discharge (31, 33, 39, 43). Although vascular endothelial damage is difficult to distinguish clinically, it can be used as a differentiating point in pathology. This is important, because the chain reaction caused by damaged endothelial cells will affect the vascular and blood system (45).

### Middle Stage

Particular attention should be paid to patients with intermediate disease who have not developed to a severe stage. The increasing viral loads infect alveolar capillary endothelial cells at the blood-air barrier through damaged alveolar epithelial cells and alveolar interstitium (21). The enhanced defense system will inevitably cause tissue damage while killing the virus. However, the overall impact of the defense system is more positive than negative. The injured vascular endothelial cells initiate the coagulation cascade system, activating coagulation factor X and promoting thrombin production. Then, this catalyzes the conversion of fibrinogen to fibrin and form pulmonary microthrombi (21, 31, 33, 39, 43–47, 61–65). As weak parts of the alveolar membrane are destroyed, blood components such as water molecules, plasma proteins, and platelets enter the alveolar cavity, forming pulmonary edema (6) (**Figure 1A**). Many histopathological findings showed that the most frequently reported morphological feature of COVID-19 disease is diffuse alveolar damage, characterized by a variable degree of edema in the exudate phase (42, 66, 67). As long as reduction of alveolar volume is compensated and thrombosis can be prevented or dissolved, the trend towards severe complications can be blocked. However, even if the criteria for discharge are met after effective treatment, the influence of impaired alveolar ventilation and pulmonary microcirculation in the course of COVID continues, and the pulmonary tissues are vulnerable to further damage (11–13, 40, 41). In another study, no significant differences in forced expiratory volume in 1s (FEV1), forced vital capacity (FVC), or their rates were observed nearly one month after discharge, regardless of the severity of COVID-19. DLCO values decreased significantly with increasing severity of clinical symptoms (total 47.2%, mild 30.4%, moderate 42.4%, severe pneumonia 84.2%) (68). Huang et al. observed that 30 days after discharge, DLCO values of patients were notably different (< 80%), 42.5% in non-critical patients versus 75.6% in severe patients (69).

### Advanced Stage

When the disease progresses to the severe/critical stage, the primary clinical task is to prevent and treat multiple organ failures and prolong life regardless of the risk of subsequent sequelae. With the exponential increase in SARS-CoV-2 particles, a large number of immune cells activate and release cytokines while gathering around and infiltrating into the lung tissue, thus initiating relevant transduction pathways and a cascade of inflammatory reactions. This creates a vicious cycle that eventually leads to a cytokine storm (70–77). Severe cases can also be accompanied by lymphocytic depletion, leading to suppression or even failure of the immune system (2, 78). Direct and rapid cytotoxic effects of plasma from critically ill patients on umbilical cord blood tubule cells were found *in vitro* (79). Researchers extracted plasma from healthy donors, non-intensive care unit (non-ICU) patients with COVID-19, intensive care unit (ICU) patients with COVID-19, and convalescent patients with COVID-19. Results showed that plasma from both COVID-19 patients and convalesced patients significantly reduced human pulmonary microvascular endothelial cells activity compared to healthy plasma, but plasma from ICU patients induced the greatest cytotoxicity. Blood vessel involvement through endotheliitis is also one of the distinguishing features of COVID-19. Microthrombi within alveolar capillaries, precapillary arteries, and postcapillary venules were frequently reported (66). Studies have shown that alveolar capillary microthrombi were 9 times more common in patients with COVID-19 than in patients with influenza, and the amount of new pulmonary vessel growth were 2.7 times higher than in patients with influenza (80). Damaged vascular endothelium contributes to a pre-thrombotic state, further activating the clotting pathway, accelerating (micro) thrombogenesis, and reducing alveolar blood flow. Impaired pulmonary vascular endothelium can cause uncontrolled activation of coagulation cascades, further leading to vascular thrombosis or fatal pulmonary symptoms of fibrosis (21) (**Figure 1B**). Many studies have suggested that severe and critical COVID-19 is associated with an increased incidence of diffuse thrombosis or pulmonary blood vessel thrombosis (2, 81–83). In severe cases, hypoxic capillary constriction and pulmonary microthrombus, thrombosis, and/or embolism cause slow blood flow, local blocked microvasculature, elevated pulmonary capillary pressure, and overall pulmonary hypertension (84). Changes in the vasculature, coupled with extensive inflammation of lung tissue, enhance the permeability of the air-blood barrier, resulting in vascular leakage with plasma and blood cells entering the alveolar cavity. Pulmonary hyaline membrane formation, acute respiratory distress, and pulmonary fibrosis exacerbate dyspnea (1–8) Impaired pulmonary vascular endothelium can cause uncontrolled activation of coagulation cascades, further leading to vascular thrombosis or fatal pulmonary symptoms of fibrosis (21) (**Figure 1C**). Therefore, besides damaged alveolar structure, reduced effective volume of the alveolar cavity, and difficulty in gas dispersion (85), (**Figure 1D**) insufficient alveolar blood flow caused by thrombus also needs timely improvement. However, severe or critical illness can present challenges. The incidence of bleeding events and sequelae is relatively high and monitoring is essential to maintain the patient’s health status (86–92). The importance of thrombus and embolic events in severe and critically ill patients is widely recognized (93–95). But the occurrence of hypoxemia even with good lung compliance in the early stage also indicates that early abnormal pulmonary blood perfusion may also exist (26, 96, 97). Autopsy results showed microthrombi in the lung but no destruction of surrounding alveolar structures (98), further suggesting that pulmonary blood perfusion is of great importance in the formation of microthrombi. Since this circumvention of traditional ARDS formation has been found, attention should also be paid to lung damage caused by microthrombi in patients recovering from COVID-19.

## Lung Injury in Long COVID

The most commonly reported lingering symptoms of COVID-19 at discharge are fatigue, muscle weakness, sleep disturbances, abnormal lung dispersion, anxiety, and depression (12). Although fatigue and weakness are the most common effects in long COVID, some survivors also report persistent severe symptoms and organ dysfunction (88). A meta-analysis of 16 cohort studies showed that discharged patients could develop residual symptoms in multiple organs, including cardiopulmonary (chest pain, dyspnea, cough, sore throat, and palpitations), nerve (dysmnesia, cognitive disorder, headache, dysgeusia, and dysosmia), gastrointestinal tract (diarrhea, vomiting, abdominal pain, and anorexia), eyes (conjunctivitis), skin (urticaria), musculoskeletal system (myalgia, and arthralgia), etc. (86) In addition to the psychological impact, there is overwhelming evidence that the lung is the most severely affected organ in COVID-19 patients, both in the progressive and convalescent stages (99, 100). In a 6-month follow-up study involving 1733 discharged patients, those requiring high flow nasal catheter (HFNC), non-invasive ventilation (NIV), or intermittent mandatory ventilation (IMV) had an odds ratio (OR) of 4.60 (after multivariable adjustment) for diffusion disorders compared with those requiring no supplemental oxygen. 36% of patients in the severest group had dyspnea with a modified Medical Research Council (mMRC) score > 1 (severe dyspnea) at six months. 50% of patients who completed high-resolution computed tomography chest scans across different severity scales had at least one CT anomaly, with GGO being the most common, followed by irregular lines (12). Revisiting the survivors after 12 months showed a slight increase in the rate of dyspnea. There was no improvement in pulmonary diffusion impairment. And the incidence of pulmonary diffusion impairment was 23% in the no oxygen group, 31% in the oxygen-required group, and 54% in the group with HFNC, NIV, or IMV. The proportion of CT abnormalities decreased significantly over time. But 76% of patients in the severe group still had GGO, and the proportion of patients with thickened interlobular septa increased significantly (11). Wu et al. tested lung function in 83 survivors of severe COVID-19 pneumonia. Although the 6mWT and dyspnea score showed significant improvement at 12 months, 33% had DLCO < 80%, and 24% had GGO radiological abnormalities (13). While most studies have focused on the long-term effects of COVID-19 on hospitalized patients, little is known about the statistics of long COVID in patients with mild or asymptomatic disease. SARS-CoV-2 infection can have subtle effects on the body, even if the patient does not require hospitalization. A study of 8,983 non-hospitalized patients two weeks after a positive test showed that these individuals had a slightly increased risk of initial diagnosis of dyspnea (1.2% vs. 0.7%) and venous thromboembolism (0.2% vs. 0.1%) compared with matched SARS-CoV-2-negative individuals. However, similar results were not found for increased risk of serious complications (such as ischemic stroke, encephalitis, psychosis, or multisystem inflammatory syndrome in children), as previously seen in severe COVID-19 hospitalizations. Positive patients were more likely to initiate bronchodilator therapy, particularly short-acting beta2 agonists (17% vs. 13%), which may be associated with dyspnea (40).

In one meta-analysis of 894 subjects from seven studies, lung function tests showed that low diffusion ability was the most common abnormality, followed by reduced lung volume, while airflow obstruction was relatively uncommon (86). Damage and repair of the blood-air barrier play an essential role in long COVID. Alveolar epithelium is a single layer of epithelial cells in which a subpopulation of alveolar type II cells undergoes self-repair after injury and act as precursors of type I cells. Alveolar type II epithelia are the dominant target cells for SARS-CoV-2. Therefore, impaired type II cells can significantly impede epithelial repair mechanisms, resulting in incomplete repair, scarring, and fibrosis (59). The initial response to the destruction of the alveolar epithelial-endothelial barrier is edematous infiltration in the alveoli and interstitial portion. This is followed by proliferation as the alveolar barrier is rebuilt by removing exudate. However, in some patients, it progresses to excessive fibrosis rather than dissipating inflammation. During the recovery of influenza and SARS, evidence of parenchymal fibrous bands and tractive bronchiectasis has been observed (101, 102). Studies have also found that elevated growth factor receptor B1 mediates extracellular interstitial protein deposition, chemotactic fibroblast migration, and the transformation of fibroblasts into myocytes (4). It has also been suggested that respiratory virus infection may induce significant fibroblast activation during convalescence (12). It is unclear whether COVID-19-associated ARDS causes irreversible pulmonary fibrosis. Studies have observed dramatic increases in the number of lung fibroblasts and collagen deposits in cases of fatal COVID-19 disease (1–8). However, whether long COVID fibrosis will stabilize and subside in subsequent years remains uncertain (5).

When injured host cells release damage-related molecular patterns, the pro-inflammatory molecules and activated immune cells lead to endothelial cell damage, hypoxia, and dysfunction in pulmonary vessels (21). Hypoxia is a driver of vascular remodeling, inducing the activation of endothelial, mesenchymal, and immune cells and promotes thrombotic fibrosis and epithelial-mesenchymal transformation in COVID-19 patients. Similar vascular remodeling can occur in pulmonary hypertension and chronic obstructive pulmonary disease, but the degree of remodeling is greater in patients with COVID-19. In COVID-19, the endothelial cells transform into smooth muscle cells. Proliferation, migration, and hypertrophy of vascular smooth muscle cells were observed at the cellular level (7). In addition, overexpression and high levels of pro-angiogenic factors (such as vascular endothelial growth factor (VEGF), hypoxia-inducible factor 1α (HIF-1α), IL-6, tumor necrosis factor receptor superfamilies 1a and 12, and angiotensin-converting enzyme 2(ACE2)) have been found in both living and dead COVID-19 patients (103). In one study, compared with healthy volunteers, patients with COVID-19 had more pulmonary vessels with a 5-30mm^2^ cross-sectional area and fewer small vessels (0-< 5mm^2^). However, there was no difference in overall lung blood volume, suggesting blood redistribution between blood vessels of different sizes (43). Histological evaluation of early COVID-19 showed an abnormal increase in the number of pulmonary blood vessels, accompanied by hyperemia, dilation, and distortion. CD4^+^ T lymphocytes infiltrated the edema wall and thickened post-capillary venules (44). Compared with the normal vascular endothelium, these new blood vessels, together with the damaged vascular endothelium, are still activated in the convalescence period. They are therefore unable to fully fulfill their role in maintaining the normal blood-air barrier in an anti-coagulant state. As a result, patients can still suffer from respiratory insufficiency and other pulmonary symptoms following COVID-19.

## New Point: PS and Thrombosis

Pulmonary vascular endothelial cells prevent thrombosis by binding to TF pathway inhibitors (TFPIs) and blocking the action of the FVIIa-TF complex (104). The presence of various endothelial injury biomarkers, including extracellular vesicles, confirms the persistence of vascular damage in convalescent COVID-19. Significantly elevated thrombin and endothelial cell biomarkers (vWF antigen (vWF: Ag), vWF propeptide (vWFpp), FVIII, and sTM) were observed in the convalescence period. At this point, most patients have normalized acute phase markers, including C-reactive protein, neutrophil counts, white blood cell counts, IL-6, and sCD25 levels (105). Persistent endothelial lesions were also observed during recovery in non-hospitalized patients (31). Impaired pulmonary vascular endothelium can cause uncontrolled activation of coagulation cascades, further leading to vascular thrombosis or fatal pulmonary symptoms of fibrosis (21). Evasio et al. monitored serological markers in 75 patients who had been discharged from the hospital for two months after COVID-19. They found high concentrations of D-dimer, and this persistent change raised the long-term risk of thromboembolic disease in convalescence patients (106).

Ongoing monitoring of COVID-19 patients after discharge from the hospital is necessary to understand the breadth and severity of long-term effects. However, COVID-19 has not existed long enough to complete large-scale cohort studies to examine its long-term impacts on infected patients in detail. Although a critical factor in the development of disease, thrombus-related indicators have rarely been comprehensively studied (106). Thrombosis is a pathological outcome of the local microenvironment. The research on thrombosis should not be limited to its subsequent influence on tissues or organs but should also include the mechanisms involved in thrombosis formation, such as the close connection with vascular endothelium, damage to blood cells, formation of the procoagulant state, and the existence of microthrombi (83, 107–110). Fibrin clumps formed in hypercoagulable conditions are difficult to detect, in contrast to thrombus formation (111). But during the transition period between disease progression and recovery period, it is difficult to judge the extent of the risk of locally developing arteriovenous thrombosis. It is also difficult to confirm whether there has been improvement of the local endothelial injury and return to an anti-coagulant state (21). However, these processes are undeniably common in COVID-19. Therefore, in long COVID, the influence of thrombosis should be assessed from the onset of the prothrombotic state. The degree of early injury and the progression from functional to organic lesions are associated with dyspnea that affects long-term quality of life.

**Figure 2:**
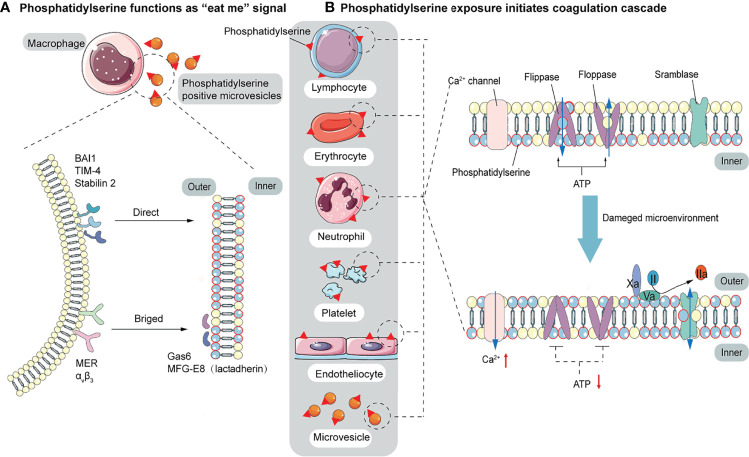
The function of PS. **(A)** PS acts as a signal to be engulfed by macrophages with two recognition modes. One is directly recognized by phagocytic receptors BAI1, TIM-4, and Stabilin 2. The other is indirect recognition. The bridging molecules MFG-E8 (also known as lactadherin) and GAS6 bind to PS, which is recognized by membrane proteins MER and α_V_β_3_. These two recognition patterns are not mutually exclusive and may co-occur. **(B)** PS provides a negatively charged surface to initiate and maintain coagulation. In normal conditions, PS is sequestered in the inner layer of cell membranes by the action of floppase and flippase. When intracellular Ca^2+^ increases, the ATP-dependent translocation enzyme is blocked, and the scramblase is activated, resulting in a random distribution of PS to both sides of the membrane. PS, initially located in the cell’s inner membrane, is exposed to the outer side. On the outer membrane, PS provides active clotting catalytic surfaces for forming TF-FVIIa complex, factor X-enzyme complex (FIXa-FVIIIa-Ca^2+^-PL), and prothrombinase complex (FXa-FVa-Ca^2+^-PL).

Elevated endothelial stress products are present in the circulating blood of COVID-19 patients. Although endothelial alterations are not specific, thrombogenesis caused by endothelial alterations in COVID-19 results in fibrin deposition in small blood vessels in the lungs and other organs. An early step in the thrombogenesis process is the expression of the pro-coagulant phospholipid PS. In normal conditions, PS is confined to the inner layer of cell membranes by the actions of floppase and flippase. When intracellular Ca^2+^ increases, the ATP-dependent translocation enzyme is blocked, and scramblase is activated, resulting in a random distribution of PS to both sides of the membrane (112). Once exposed on the outer membrane, PS mediates TF decryption and activation, initiating the coagulation cascade (113). PS also provides an active catalytic surface for the formation of the TF-FVIIa, factor X-enzyme (FIXa-FVIIIa-Ca^2+^-PL), and prothrombinase (FXa-FVa-Ca^2+^-PL) complexes, leading to the conversion of fibrinogen to fibrin. Pulmonary microthrombi can further develop into pulmonary arteriovenous thrombosis and decrease alveolar blood flow (**Figure 2B**). In addition to providing a negatively charged surface to initiate and maintain clotting functions, PS also acts as a signal to be engulfed by macrophages, avoiding the activation of inflammation and autoimmunity (58). There are two modes of recognition between macrophages and PS-expressing cells. One is direct recognition by the phagocytic receptors: brain-specific angiogenesis inhibitor 1 (BAI1), T cell immunoglobulin mucin 4 (TIM-4), and Stabilin 2. The other is indirect recognition. The bridging molecules milk fat globule epidermal growth factor 8 (MFG-E8, also known as lactadherin) and growth arrest-specific 6 (GAS6) bind to PS (114, 115), which is recognized by membrane proteins MER and α_V_β_3_. These two recognition patterns are not mutually exclusive and may co-occur (57) (**Figure 2A**). Biomarkers of platelet activation are associated with thrombosis and mortality risk in COVID-19 (116, 117). Several studies have pointed to microvesicles (EVs) and platelet-derived microvesicles (pEVs) as potential biomarkers in COVID-19. Elevated levels of circulating pEVs have been observed in patients with SARS-CoV-2 infection and significantly elevated levels of pEVs in patients with severe disease (118–122). One study using flow cytometry of patient samples found that the frequency of PS^+^ cells in the blood of all COVID-19 patients within a week of diagnosis was considerably higher than that of peripheral blood mononuclear cells (PBMC) from healthy or recovered donors. The number of PS^+^ PBMC is strongly correlated with the severity of disease and can better predict the need for respiratory support (123). Corresponding autoantibodies to the PS/prothrombin complex have also been found in COVID-19 patients (124). EVs, approximately 100 to 1000 nm in diameter, are produced by budding and shedding of the plasma membrane of various blood cells. Since EVs are unable to maintain membrane asymmetry, they are characterized by PS externalization and can affect the regulation of coagulation and inflammation (125–127). In both sepsis and COVID-19, upregulation of PS exposure can occur on cell surfaces (including endothelial cells, platelets, red blood cells, neutrophils, and lymphocytes) and extracellular particles (128–130). Karina et al. reported significantly increased depolarization of mitochondrial inner transmembrane potential and cytosolic Ca^2+^ and PS externalization in ICU patients compared with healthy controls and non-ICU patients with COVID-19 (131). Since the localization of PS on subcellular organelles was first reported in 2008, we have been focusing on PS-induced hypercoagulability and thrombotic events. The presence of PS^+^ blood cells, endothelial cells, and particles has been found in experimental studies of acute promyelocytic leukemia, nephrotic syndrome, sepsis, inflammatory bowel disease, acute stroke, and triple-negative breast cancer, suggesting that PS-induced procoagulant activity may be common in various diseases (132–136).

## Inhibiting the Prothrombotic State

In terms of thrombosis, thrombogenesis should be blocked from the beginning, and the procoagulant state should be alleviated to reduce the incidence of sequelae. D-dimer is commonly used as a marker for thrombosis, and a higher D-dimer level is independently associated with a higher risk of death. In some studies, changes in D-dimer level are used to distinguish the severity of COVID-19 in the middle and late stages (137–140). Because existing anti-thrombotic interventions appear to have limited effects in the severe and critical stage, it is crucial to take measures at moderate or even mild stage to improve patient outcomes and reduce the occurrence of sequelae. In late stage COVID-19 patients, the maximum solubility assessed by rotary thromboelastometer analysis was significantly less in patients with thrombus compared with patients without thrombotic events (141–143). Meanwhile, the increased levels of tissue plasminogen activator and plasminogen activator inhibitor-1 in patients’ blood circulation further suggest impaired fibrinolysis (144). It is important to relieve thrombus formation tendency or remove (micro) thrombi early. Therefore, the focus should be on early antithrombotic therapy, including anticoagulation, anti-platelet activation, and thrombolytic therapy as appropriate.

As a means to prevent thrombosis and relieve hypercoagulability, anticoagulant therapy has been studied primarily in the acute stage of COVID-19. Currently, all guidelines agree that low-molecular-weight heparin thromboprophylaxis should be used in all hospitalized patients with COVID-19, recognizing that hypercoagulability can contribute to more severe disease progression (145–149). The commonly used anticoagulant drugs are low molecular weight heparin (LMWH) (such as enoxaparin) and direct oral anticoagulants (such as Rivarxaban and dabigatran). Heparin can also play a part in controlling leukocyte migration and complement activation (150). Treatment with high-dose prophylactic anticoagulation was associated with a significantly reduced risk of pulmonary embolism (hazard ratio, 0.72, 95%CI, 0.53-0.98) in a study of patients admitted to ICU 14 days after COVID-19 diagnosis (151). A New York study, using the Cox proportional risk model to assess the effect of therapeutic-dose anticoagulation on in-hospital mortality, found that patients treated with anticoagulant had a 22.5% in-hospital mortality and a median survival of 21 days, compared with 22.8% who did not receive anticoagulant and a median survival of 14 days. Systemic therapeutic dose anticoagulation may be associated with improved outcomes in hospitalized patients with COVID-19 (152). One study found that for non-critical patients with COVID-19, therapeutic anticoagulation improved hospital discharge survival without organ support (153). Theoretically, inhibition of hypercoagulability can block the occurrence of microthrombotic events, especially with early treatment. As the site of initial infection, lungs are the most susceptible organ where the effects of inflammation and thrombosis appear early. With early anticoagulation, the pulmonary circulation remains unblocked so that inflammatory substances formed in the lungs that enter systemic circulation can be removed by the immune system. Limiting the level of inflammation in the lungs can reduce the damage to the alveoli and prevent the generation of cytokine storms and PS storms. Effective control of inflammation and improvement of hypoxia reduces damage to endothelial cells and prevents a large number of endothelial cells from transitioning to a defensive state.

Some studies have failed to show prolonged survival time or improved survival rate as a result of anticoagulant therapy. In these studies, most samples are patients with severe or critical disease. A large number of clots have formed, resulting in the depletion of clotting factors resulting in a low fibrinolytic state. Under these conditions, anticoagulants do not protect the patient (138, 154). After the necessary thrombolysis or thrombectomy to narrow and remove the clot, the alveolar perfusion blood flow is improved, and function is gradually restored. However, the inflammatory storm and PS storm, which were originally confined to the lung, can also quickly enter the systemic circulation, accelerate injury to the extrapulmonary organs, and even lead to death. Another possible explanation is the dosage of anticoagulant therapy. Some studies have used therapeutic doses of LMWH for thromboprophylaxis in critically ill patients with thrombotic risk factors (146). The incidence of bleeding events with therapeutic dose anticoagulants was 3.0%, and 1.7% with the prophylactic dose. Among all bleeding events, the gastrointestinal tract was the most common (50.7%), followed by mucosa (19.4%), bronchia (14.9%), and intracalvarium (6%), but fatal bleeding events were rare (155–157). This suggests that therapeutic dose anticoagulants improve overall survival. Meanwhile, some studies have shown that therapeutic doses do not increase the risk of bleeding (158). However, some studies have avoided an increased dose of thromboprophylaxis due to a slight increase in bleeding events (159, 160). The difficulty of using anti-thrombotic therapy during the period of severe and critical illness highlights the importance of timely comprehensive antithrombotic therapy before the late stage. From this perspective, treatment to prevent mild or moderate disease from progressing to a severe or critical condition will significantly improve the overall prognosis. Temporary appropriate comprehensive treatment can effectively block the trend of severe disease development and reduce the occurrence of sequelae in the long run (161). In a prospective study in France, analysis using propensity score matching confirmed that pre-hospital anticoagulant treatment was associated with a better outcome, with a risk of 0.43(95% CI, 0.29-0.63) for admission to intensive care (162). The difference in therapeutic anticoagulant efficacy between moderate and critically ill patients may be attributed to severe inflammatory responses, when thrombotic complications in critically ill patients are too pronounced to recover. In non-ICU patients, therapeutic anticoagulant therapy may still help maintain an appropriate balance (163). As for the risk of bleeding events, because the vascular endothelial cells are relatively undamaged and the coagulation factors are not yet depleted, the risk of antithrombotic bleeding is lower than the risks associated with respiratory distress syndrome, multiple organ failure, and/or sequelae. Christopher T Rentsch and colleagues found that patients who received prophylactic anticoagulation within 24 hours of admission had a 27% reduction in 30-day mortality (hazard ratio, 0.73; 95% confidence interval, 0.66 to 0.81) compared with those who did not. Receiving prophylactic anticoagulant therapy was not associated with an increased risk of bleeding requiring transfusion (hazard ratio, 0.87,0.71 to 1.05) (164). Unobstructed blood flow ensures adequate blood perfusion to the alveoli, reducing hypoxemia incidence. Unimpeded pulmonary circulation slows or prevents the leakage of plasma into the alveolar cavity, which is aggravated when there is a large pressure difference between the two sides of the alveolar membrane. Thus improved circulation prevents pulmonary hypertension, lung hyaline membrane formation, and lung consolidation, thereby reducing the risk of death and sequelae. Current guidelines have no routine precautions for discharged patients. Some recommend anticoagulant prophylaxis (LMWH or direct oral anticoagulants) in high-risk patients with a low risk of bleeding (165).

The application of antiplatelet drugs mainly includes aspirin (cyclooxyganese inhibitor), clopidogrel (P2Y12 inhibitor), or dipyridamole (adenosine deaminase and phosphodiesterase) (166, 167). It has been reported that clopidogrel may interact with antiviral drugs and should be used with caution. Dipyridamole may be considered for antiplatelet therapy in the presence of renal insufficiency to reduce bleeding due to drug build-up. Aspirin is the most commonly used antiplatelet drug with anti-inflammatory, antipyretic, analgesic and antiplatelet functions. In infectious diseases, aspirin is associated with a reduction in thrombotic inflammation, clinical complications, and in-hospital mortality (168). In a retrospective cohort study of COVID-19, aspirin had some benefits in reducing the risk of mechanical ventilation, ICU admission, and in-hospital mortality (169). Similar results were found in a small observational cohort study of adults with COVID-19 when aspirin was taken at least seven days before or within 24 hours of hospitalization compared with no aspirin (170). In another recent observational study, 730 patients who received antiplatelet therapy had lower mortality and shorter mechanical ventilation duration during hospitalization than 6986 patients who did not receive antiplatelet treatment (171). However there are clinical trials showing that aspirin is not associated with a reduced risk of ARDS (172). Aspirin, as an irreversible platelet inhibitor, is cheap and available, and trials are investigating its effect on the risk of thrombosis.

## PS: Novel Therapeutic Targets

PS, an initial factor of the coagulation cascade, could potentially be used as a new therapeutic target. Lactadherin combined with PS is a more targeted “eat me” signal that could prevent or reduce hypercoagulability (173). PS^+^ EVs and cells provide a platform for the anchoring of coagulation factors. Annexin V and lactadherin interrupt coagulation cascades by selectively binding PS (174). Lactadherin is structurally homologous to FVIII and FV and effectively blocks the availability of PS for coagulation reactions (175). The levels of PS^+^ cells and pEVs in COVID-19 patients were higher than those in healthy controls and are positively correlated with the severity of the disease. Due to the high likelihood of diffuse microthrombi and arteriovenous thrombus in critically ill patients and the procoagulant role of PS, we think that Annexin V or lactadherin could reduce the incidence of thrombosis in COVID-19 patients (118, 123). In the presence of a large amount of PS, the inhibition of upstream FXIa, FXIIa, and FXa generation can block IIa generation and avoid the formation of thrombus. While the anticoagulant effect of lactadherin has been confirmed *in vitro*, it’s anticoagulant effect *in vivo* requires additional study (176). As an under-recognized hemostatic regulator, lactadherin is a potential therapeutic agent in preventing COVID-19 thrombosis (177).

## Discussion

Due to the high prevalence of respiratory failure and the need for mechanical ventilation in COVID-19, a significant number of patients will be at risk of long-term complications following severe lung disease. Currently, the world has limited knowledge of long-term lung disease in survivors, and long COVID is still a public health concern. There may be variation in the phenotypes of long COVID, and different post-infection states exist in survivors of COVID-19 ARDS. Many people continue to experience respiratory symptoms for months after an acute infection, especially those with underlying asthma. Other subgroups of patients appear to worsen within three to four weeks of initial infection and brief recovery. Some patients start with a very mild course of illness (do not require medical care or hospitalization) but go on to develop infectious symptoms and ARDS after a few weeks. Thrombus is a crucial factor in the progression of COVID-19 to severe disease, and its importance in long COVID has not been fully assessed. Early treatment of microthrombi can reduce not only mortality but also reduce the incidence of sequelae. PS expression is common when cells are damaged or undergoing apoptosis. And epidemic diseases often have a large number of cell damage and death, forming PS storms and promoting thrombosis. While vaccines are essential in preventing severe disease, effective treatment of COVID-19 remains essential given the rise of virus variants and the waning effectiveness of vaccines.

## Author Contributions

MX prepared figures and wrote the manuscript. HJ and CW provided helpful comments and acquired data. JS came up with the project, designed the study, contributed to successive drafts, and reviewed this manuscript. VN gave the revision advice and polished this review. All authors read and approved the final manuscript.

## Conflict of Interest

The authors declare that the research was conducted in the absence of any commercial or financial relationships that could be construed as a potential conflict of interest.

## Publisher’s Note

All claims expressed in this article are solely those of the authors and do not necessarily represent those of their affiliated organizations, or those of the publisher, the editors and the reviewers. Any product that may be evaluated in this article, or claim that may be made by its manufacturer, is not guaranteed or endorsed by the publisher.
